# The Immunomodulatory Effects of Phellodendri Cortex Polysaccharides on Cyclophosphamide-Induced Immunosuppression in Mice

**DOI:** 10.1155/2021/3027708

**Published:** 2021-11-24

**Authors:** Yi Cheng, Lu Liu, Simei Mo, Jianxiong Gao, Hongjian Zhang, Heli Zhang, Chunsheng Zhang, Xu Song, Lixia Li, Zhe Geng

**Affiliations:** ^1^Department of Physical Education, Chengdu University of Information Technology, Chengdu, China; ^2^College of Veterinary Medicine, Sichuan Agricultural University, Chengdu, China

## Abstract

Cyclophosphamide is a commonly used anticancer drug, and immunosuppression is one of the most common side effects. How to recover the immunological function is important for cyclophosphamide-treated patients. In the present study, Phellodendri Cortex polysaccharides (CPP) could enhance the proliferation of mouse spleen lymphocytes in vitro. The immunoregulatory function of CPP was then investigated in cyclophosphamide-induced immunosuppressed mice. In CPP-treated groups, mice were orally treated with CPP at doses of 1, 0.5, and 0.25 g/kg bodyweight from 1 to 11 d, respectively. The cyclophosphamide was administrated in CPP and cyclophosphamide groups from 12 to 14 d. In the cyclophosphamide and normal control groups, the mice received equal volume of saline from 1 to 14 d. The results showed that CPP (1 g/kg) could significantly increase the bodyweight of mice, even during cyclophosphamide treatment. The organ coefficients of the spleen and thymus were recovered by CPP treatment. CPP upregulated the contents of cytokines (IL-2, IL-6, IFN-*γ*, and TNF-*α*) in serum, which were downregulated by cyclophosphamide. The mRNA levels of these cytokines were also elevated by CPP treatment in the spleen. Cyclophosphamide upregulated the expressions of NF-*κ*B p65, TLR4, and MyD88, suggesting that the NF-*κ*B signaling pathway was activated by cyclophosphamide. After CPP treatment, it was recovered to normal level. These results indicated that CPP alleviated the cyclophosphamide-induced immunosuppression.

## 1. Introduction

Phellodendri Cortex (“Huangbo” in Chinese), a traditional Chinese herbal, is derived from *Phellodendron chinense* Schneid. or *Phellodendron amurense* Rupr. (family Rutaceae) and known as Phellodendri Cortex Chinensis (CPC) and Phellodendri Cortex Amurensis (CPA), respectively [1]. Traditionally, it was used to clear heat, dry dampness, purge free, and remove toxicity [2]. Besides, modern pharmacological research studies indicated that Phellodendri Cortex exhibited various pharmacological activities, such as relaxing airway smooth muscle [2], inhibiting diabetes and gout [3], antioxidant [4], anti-inflammation [5–7], antiulcer [8], antibacteria, neuroprotective [9], antiviral, antidiarrhea [10], bone growth-stimulation, and immune-stimulation activities [11, 12].

Cyclophosphamide, an alkylating agent, is widely used for cancer treatment, such as ovarian, lung, and breast cancers [13]. Cyclophosphamide can induce immunosuppression which is one of the most common side effects; thus, it is also used widely in organ transplantation and autoimmune disease [14]. The immunosuppression induced by cyclophosphamide or its metabolite could affect both cell-mediated and humoral immunity [15]. The immune system is closely relevant to host's somatic functions, such as aging [16], mental state [17], inflammation [18], and various infections [19–21], which consists of different cell populations [16]. Once host suffers from infection or inflammation, the immune system protects the host from external invaders or stimulations and altered or modified internal factors [22, 23]. Therefore, how to recover the immune function under immunosuppressive status is important for keeping healthy, especially for chemotherapeutically treated patients.

Previously, Phellodendri Cortex polysaccharides (CPP) exhibited potent antitumor activity against sarcoma [24]. The CPP fraction showed marked B-lymphocyte-stimulating activity [11]. However, few studies were conducted to evaluate immunoenhancement activity of CPP under immunosuppression. In the study, the immune-stimulation activity of CPP was investigated in immunosuppressive mice induced by cyclophosphamide for developing a new herbal-derived immunostimulant.

## 2. Materials and Methods

### 2.1. Extraction and Purification of Polysaccharides

The Phellodendri Cortex (batch number: 20200607) was purchased from Sichuan Zhongyong Pharmaceutical Co., Ltd. (Chengdu, China). The dried Phellodendri Cortex powder (200 g) was defatted by ethanol at 75°C for 6 h, and then, the defatted powder was decocted in distilled water for 3 times (1 : 10, g/v). The decoction was collected and concentrated, and then, ethanol was added (v/v, 1 : 3). The supernatant was removed by centrifugation, and the sediment (total polysaccharides) was collected. The crude polysaccharides were dissolved in hot water and deproteinized with Sevag's reagent, followed by dialysis against distilled water for 3 d to remove other impurities. Finally, CPP (8.27 g) was obtained by lyophilization, and the extraction rate was 4.14%. The content of CPP was 88.58%, which was detected by the phenol-sulfuric acid method. In the following tests in cells, the CPP was dissolved in phosphate-buffer saline (PBS) (4 mg/mL) and then diluted with culture medium. For the animal study, CPP was dissolved in saline.

### 2.2. Ethics Approval and Animals

The experimental protocol was approved by the National Institute of Ethics Committee at Chengdu University of Information Technology (approval number 2020–053). Four-week-old specific pathogen-free (SPF) KM mice (20 ± 2 g) were commercially provided by Chengdu Dossy Experimental Animals Co., Ltd. (license no. SCXK (Sichuan) 2015–030). The mice were fed normally with sterilized mice maintenance feed (Chengdu Dossy Experimental Animals Co., Ltd., China) at controlled temperature (23 ± 2°C), humidity of 55 ± 5%, and a 12 h light-dark cycle. After acclimatization for 7 d, the mice were used for the following study.

### 2.3. Preparation of Mice Splenic Lymphocytes

The normal and immunosuppressed (induced by cyclophosphamide) mice were sacrificed and sterilized in 75% (v/v) ethanol for 5 min and then dissected in clean bench. The spleen was separated and steeped in PBS. Cell strainers (Corning, USA) were used to grind spleens with rubber stopper syringes. The cell suspension was centrifuged at 1200 ɡ at 4°C for 15 min, and the cell pellet was resuspended in PBS. Erythrocytes were lysed by lysis buffer for 10 min, and then, the lysate was centrifuged at 1200 ɡ at 4°C for 10 min. The cell pellet was resuspended with RPMI-1640 supplemented with 10% fetal bovine serum (Gibco), 100 U/mL penicillin, and 100 *μ*g/mL streptomycin. The cells were kept at 37°C and 5% CO_2_ for 4 h and then used for the following study.

### 2.4. Spleen Lymphocytes Cytotoxicity Assay

The cytotoxic effect of CPP on lymphocytes was measured by Cell Counting Kit-8 (CCK-8) assay. Cells were treated with different doses of CPP (400, 200, 100, and 50 *μ*g/mL), respectively. After incubation at 37°C for 48 h, 10 *μ*L of the CCK-8 solution (CA1210; Solarbio, Beijing) was added to each well. The plates were reincubated at 37°C for 30 min, followed by measurement of absorbance values at 450 nm using a microplate reader (Bio-Rad, USA). The cell viability was calculated as follows: cell viability = OD450 nm values of cells with CPP treatment ÷ OD450 nm values of control cells without treatment.

### 2.5. Spleen Lymphocytes Proliferation Assay

The proliferation promotion effect of CPP on lymphocytes was detected with CCK-8 assay. 100 *μ*L splenocyte (5×106 cell/mL) was added into each well. The cells were divided into 4 groups: CPP (200 *μ*g/mL), LPS (2 *μ*g/mL) + CPP (200 *μ*g/mL), LPS (2 *μ*g/mL), and control. The control group received an equal volume of culture medium. There were 8 repeats in each group, and the cells were cultured at 37°C with 5% CO_2_ for 48 h. The CCK-8 solution (10 *μ*L) was added into each well for another incubation for 4 h. The optical density (OD) of each well was measured at 450 nm. The cell viability = OD450 nm values of cells with treatment ÷ OD450 nm values of control cells.

### 2.6. Experimental Design

50 mice (20 ± 2 g) were divided into 5 groups randomly (*n* = 10). The groups included the high dose of CPP (1 g/kg) group, medium dose of CPP (0.5 g/kg) group, low dose of CPP (0.25 g/kg) group, cyclophosphamide group, and control group. From 1 to 11 d, the CPP groups were orally administrated with CCP. The CY group and control group received equal volume of saline. From 12 to 14 d, the CCP groups and CY group received cyclophosphamide (20 mg/kg) by intraperitoneal injection; meantime, the mice in the CCP groups orally administrated with different doses of CCP and control group received equal volume of saline by gavage. The bodyweight variation of each mouse was recorded from 1 to 14 d. At 14 d, all mice were subject to euthanasia, and then, the spleen and thymus in each mouse were collected and weighed. The organ coefficient of each mouse was calculated according to the following formula: organ coefficient (mg/g) = organ weight/bodyweight.

### 2.7. Serum Cytokines Assay

Blood was sampled by eyeball extirpating in each group. After coagulation at room temperature for 30 min, the blood was centrifuged at 3000 ɡ for 5 min. The serum in each group was collected for cytokines assay (IL-2, IL-6, IFN-*γ*, and TNF-*α*) with ELIAS kits according to the manufacturer's instructions (Beijing Gersion Biotechnology Co., Ltd, China).

### 2.8. Transcriptional Levels of Target Genes Assay

The mRNA levels of cytokines (IL-2, IL-6, IFN-*γ*, and TNF-*α*) in the spleen were evaluated by real-time PCR (RT-PCR) assay. In brief, total RNA from the spleen in each group was extracted with RNAiso Plus (no. 9108, TaKaRa, China) according to the instructions of manufacturer. Equal amounts of RNA samples were reverse-transcribed into cDNA with RevertAid First Strand cDNA kit (no. K1622; Thermo Scientific^TM^) according to manufacturer's protocol. The RT-PCR was performed with SYBR Green Supermix kit (Bio-Rad, USA), and primers are given in Table 1. The PCR cycling was 3 min at 95°C and then 40 cycles of 10 s at 95°C, 30 s at 60°C, and finally 55°C for 5 s. In the end, the melting curve analysis was conducted. The difference in each sample was normalized with the expression of *β*-actin. Data analysis were performed by the Bio-Rad CFX Manager software (Bio-Rad, USA).

### 2.9. Western Blotting

The expressions of NF-*κ*B p65, TLR4, and MyD88 were detected by Western blotting. The total proteins of the spleen were extracted with a protein extraction kit (BOSTER, Wuhan, China). Total spleen lysates were run on a 10% sodium dodecyl sulfate-polyacrylamide gel electrophoresis (SDS-PAGE) gels for 30 min with 80 V and 70 min with 120 V and then transferred to polyvinylidene difluoride (PVDF) membranes (Millipore, USA) for 90 min with 200 mA. The membranes were blocked with 5% BSA for 90 min (Solarbio, China) at room temperature, and then, proteins were stained using primary antibodies directed against TLR4 (CST, 14358S, 1 : 1000), MyD88 (CST, 4283S, 1 : 1000), p65 (CST, 8242T, 1 : 1000), and *β*-actin (Boster, China, 1 : 1000) at 4°C overnight. The membranes were washed with Tris-buffered saline containing 0.1% Tween 20 (TBST) for 4 times and incubated with horseradish peroxidase-conjugated secondary antibody (Boster, China, 1 : 5000) for 1 h at room temperature, followed by washing for 4 times. Finally, the proteins were visualized using enzymatic chemiluminescence (ECL) reagents (Bio-Rad, USA) and normalized with the expression of *β*-actin. The ratios of protein band intensity were obtained with ImageJ software (Version 1.47, NIH, USA).

### 2.10. Statistical Analysis

All data were presented as mean ± standard deviation (SD), whose statistical significance was compared by one-way analysis of variance (ANOVA) with Duncan's multiple range test in SPSS 19.0 (IBM Corp., Armonk, NY, USA). *P* < 0.05 was considered statistically significant.

## 3. Result

### 3.1. The Cytotoxicity of CPP

**Figure 1:**
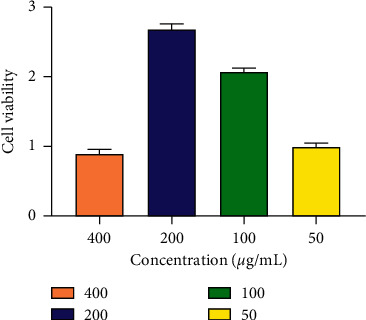
The cytotoxicity of CPP on lymphocyte. The lymphocytes from mice were treated with different doses of CPP (400, 200, 100, and 50 *μ*g/mL) for 48 h. The cell viability was measured by CCK-8 assay and calculated as follows: cell viability = OD450 nm values of cells with CPP treatment ÷ OD450 nm values of control cells without treatment. The results are shown as mean ± SD, *n* = 6.

The cytotoxic effect of CPP on lymphocytes is shown in Figure 1. The CPP at the concentration of 400 *μ*g/mL inhibited the viability of lymphocyte slightly, while CPP at concentrations of 200, 100, and 50 *μ*g/mL could promote the viability of lymphocyte.

### 3.2. The Promotion Effect of CPP on Lymphocyte Proliferation

**Figure 2:**
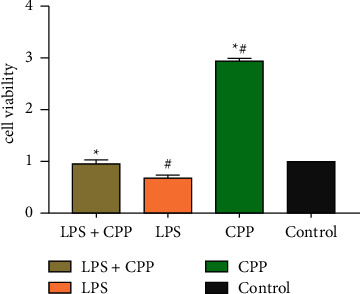
The proliferation promotion effect of CPP on lymphocytes. The lymphocytes were treated with LPS (2 *μ*g/mL) in the presence or absence of CPP (200 *μ*g/mL) for 48 h. The cell viability was measured by CCK-8 assay and calculated as follows: cell viability = OD450 nm values of cells with treatment ÷ OD450 nm values of control cells without treatment. The results are shown as means ± SD, *n* = 8. ^∗^*P* < 0.05 vs. the LPS group. ^#^*P* < 0.05 vs. the control group.

As shown in Figure 2, compared with the control group, LPS treatment could significantly decrease the viability of lymphocytes isolated from both normal and immunosuppressed mice (*P* < 0.05), and CPP treatment (200 *μ*g/mL) upregulated the viability of lymphocytes to the normal level (*P* < 0.05). Without LPS stimulation, CPP treatment could significantly promote the lymphocyte proliferation from both normal and immunosuppressed mice in comparison with untreated lymphocytes (*P* < 0.05).

### 3.3. Bodyweight

**Figure 3:**
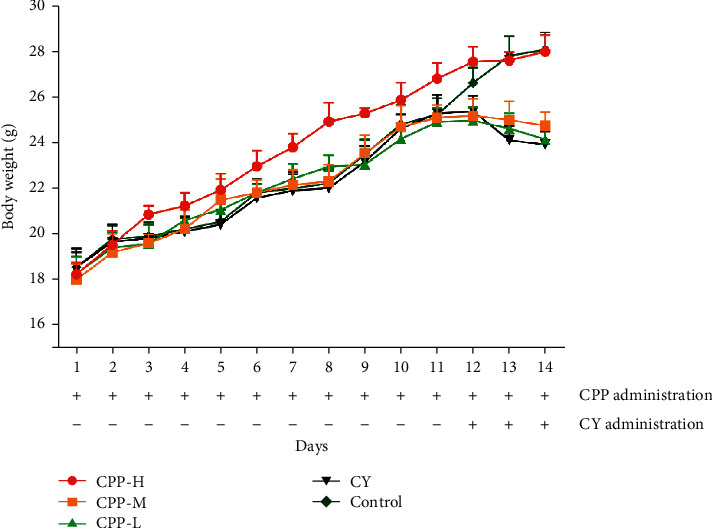
Bodyweights of mice in each group. The bodyweight of each mouse was recorded from 1 d to 14 d. CPP-H, CPP-M, and CPP-L represent the high (1 g/kg), medium (0.5 g/kg), and low (0.25 g/kg) doses of CPP groups. CY, cyclophosphamide. Control, the normal group without any treatment. The results are shown as mean ± SD, *n* = 10.

Bodyweight of mice in each group were weighed and recorded every day. As shown in Figure 3, bodyweights of mice increased gradually in each group from 1 d to 11 d. In the CPP-H group (1 g/kg), the bodyweights of mice were higher than those in the other groups, suggesting that CPP could enhance the bodyweight. From 12 to 14 d, mice except the control group received cyclophosphamide, and then, the bodyweight showed a decrease trend, especially the bodyweights of mice in the CY group decreased significantly. The mice treated with CPP at a dose of 1 g/kg could maintain the bodyweight to the level of the control group during cyclophosphamide administration.

### 3.4. Organ Coefficient

**Figure 4:**
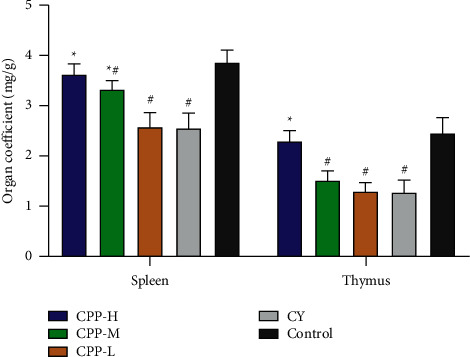
Organ coefficients of mice in each group. CPP-H, CPP-M, and CPP-L represent the high (1 g/kg), medium (0.5 g/kg), and low (0.25 g/kg) doses of CPP groups. CY, cyclophosphamide. Control, the normal group without any treatment. The results are shown as mean ± SD, *n* = 10. ^∗^*P* < 0.05 vs. the CY group. ^#^*P* < 0.05 vs. the control group.

The spleen and thymus are common immune organs, and the organ coefficients (the ratio of organ weight to bodyweight) reflect host's immune state to some extent. As shown in Figure 4, organ coefficients of the spleen and thymus in the CY group were significantly lower than those in the control group (*P* < 0.05). After CPP treatment (1 g/kg), the organ coefficients of the spleen and thymus were recovered to the level of the control group, which were significantly higher than that of the CY group (*P* < 0.05).

### 3.5. The Contents of Serum Cytokines

**Figure 5:**
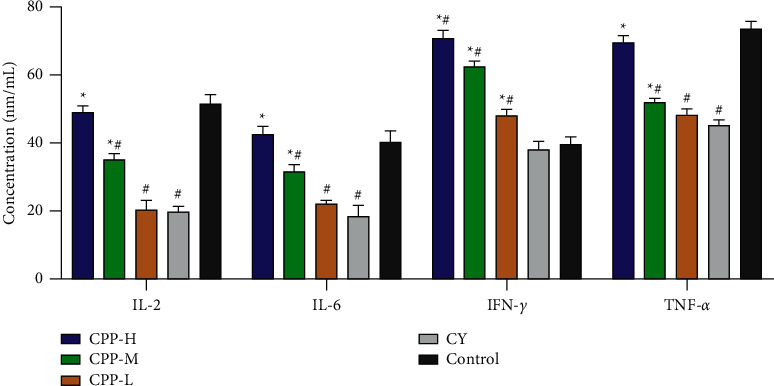
The contents of serum cytokines. CPP-H, CPP-M, and CPP-L represent the high (1 g/kg), medium (0.5 g/kg), and low (0.25 g/kg) doses of CPP groups. CY, cyclophosphamide group. Control, the normal group without any treatment. The results are shown as mean ± SD, *n* = 10. ^∗^*P* < 0.05 vs. the CY group. ^#^*P* < 0.05 vs. the control group.

Cytokines are always relative to immunoreaction, which reflects host's immune status. Therefore, the contents of IL-2, IL-6, IFN-*γ*, and TNF-*α* in serum were detected by ELISA assay. As shown in Figure 5, cyclophosphamide significantly decreased the contents of IL-2, IL-6, and TNF-*α* in serum when compared with the control group (*P* < 0.05), while CPP treatment significantly increased the contents of IL-2, IL-6, TNF-*α*, and IFN-*γ* in comparison with the CY group (*P* < 0.05). The IFN-*γ* contents in the three CPP groups were significantly higher than that of the control group (*P* < 0.05).

### 3.6. The mRNA Levels of IL-2, IL-6, IFN-*γ*, and TNF-*α* in the Spleen

**Figure 6:**
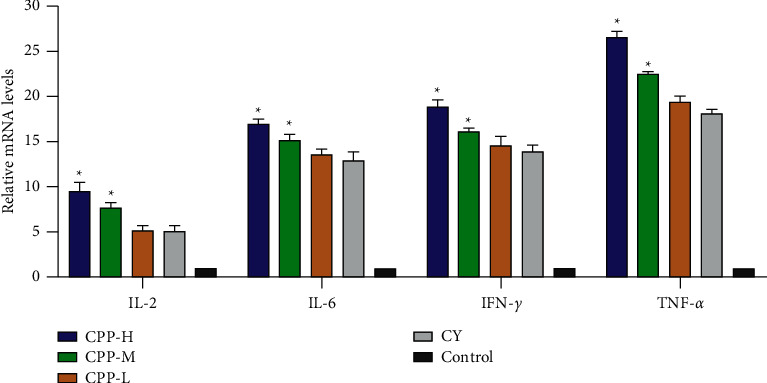
The mRNA levels of IL-2, IL-6, IFN-*γ*, and TNF-*α* in the spleen. CPP-H, CPP-M, and CPP-L represent the high (1 g/kg), medium (0.5 g/kg), and low (0.25 g/kg) doses of CPP groups. CY, cyclophosphamide group. Control, the normal group without any treatment. The results are shown as mean ± SD, *n* = 6. ^∗^*P* < 0.05 vs. the CY group.

As shown in Figure 6, compared with the control group, cyclophosphamide treatment significantly increased the mRNA levels of IL-2, IL-6, IFN-*γ*, and TNF-*α* (*P* < 0.05). CPP treatment (1 and 0.5 g/kg) significantly increased the mRNA levels of IL-2, IL-6, IFN-*γ*, and TNF-*α*, when compared with the CY group (*P* < 0.05). These results suggested that CPP could promote the expressions of IL-2, IL-6, IFN-*γ*, and TNF-*α* in the spleen of cyclophosphamide-treated mice.

### 3.7. The Effect of CPP on NF-Κb and TLRs Signaling Pathways

**Figure 7:**
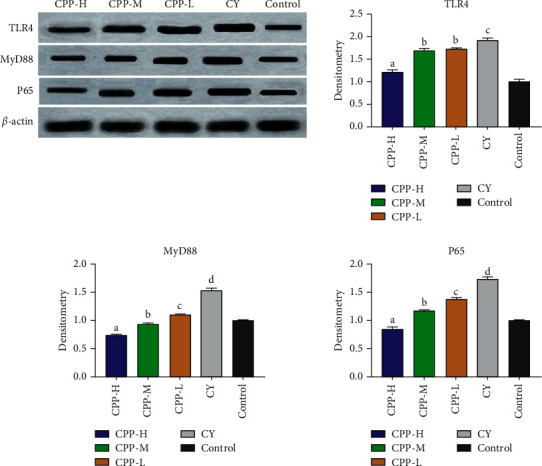
The effects of CPP on NF-*κ*B and TLRs signaling pathways in the spleen. CPP-H, CPP-M, and CPP-L represent the high (1 g/kg), medium (0.5 g/kg), and low (0.25 g/kg) doses of CPP groups. CY, cyclophosphamide group. Control, the normal group without any treatment. The results are shown as mean ± SD, *n* = 3. The different letters on a column differ significantly, *P* < 0.05.

The NF-*κ*B and TLRs signaling pathways were measured via Western blotting assay. As shown in Figure 7, the expressions of TLR4, MyD88, and p65 were significantly enhanced by cyclophosphamide treatment when compared with the control group, suggesting that NF-*κ*B and TLRs signaling pathways were activated by cyclophosphamide. While CPP treatment alleviated this trend, especially in the CPP-H group, the expressions of p65, TLR4, and MyD88 signaling pathways were significantly inhibited (*P* < 0.05).

## 4. Discussion

Since 1950s, the pharmacological activities of polysaccharides have been explored gradually. Plant polysaccharides exhibited various functions, such as immunoregulatory [25], antioxidant [26, 27], antitumor [25, 28], anti-inflammation [29, 30], antiviral [31, 32], and antiradiation effects [33]. Many studies indicated that plant polysaccharides are relatively nontoxic without significant side effects [34]. For the clinical application, the immunoregulator is necessary, while toxicity and side effect of the immunoregulator are inevitable [34]; therefore, plant polysaccharides are the best candidate drugs to regulate immunity.

In the present study, the immunoregulation of CPP was first investigated in vitro and found that CPP could promote mouse lymphocyte proliferation. LPS is an endotoxin from Gram-negative bacteria that produces a variety of biological activities in human and other animal cells [35]. It was shown that LPS can induce an immune response in normal animal bodies, which can not only regulate the distribution of B lymphocytes in the spleen but also reduce the proliferation of T lymphocytes [36–38]. Our study found that CPP promotes spleen lymphocyte proliferation and inhibits the regulatory effect of LPS on lymphocyte proliferation. Besides, the polysaccharides from *Eupolyphaga Sinensis* Walker and sulfated Chinese yam polysaccharides could also promote splenic lymphocyte proliferation [39,40].

For further confirming immunomodulatory effects, an in vivo study was performed on cyclophosphamide-induced immunosuppression mice. Immunosuppression can be achieved via various ways, such as glucocorticoids which could make organism immunosuppression via decreased chemotaxis, vessel wall permeability, and antigen phagocytosis. Cytostatics, such as purine analogs including azathioprine, cyclophosphamide, and methotrexate, could also cause immunosuppression though inhibition of DNA synthesis or inosine monophosphate dehydrogenase to impair mitosis. The calcineurin inhibitors control the transcription of interleukins in T lymphocytes via binding to intracellular immunophilins to decrease IL-2 production and T cell proliferation, including cyclosporin A, tacrolimus, and mTOR inhibitors. The use of these immunosuppressors results in severe side effect for the organism. Therefore, the proper way to build the immunocompromised model was vital and essential for the present study. Considering that cyclophosphamide was widely used to build the immunocompromised mouse model and the methods are mature, in this study, cyclophosphamide (20 mg/kg) was used as an immunosuppressor to build the immunocompromised mouse model. Changes in bodyweight and organ coefficients can reflect whether the body is in a pathological state [41]. After cyclophosphamide administration, the bodyweight and organ coefficients of the spleen and thymus decreased significantly, suggesting that the immunocompromised model was successively established. Previous studies have found that the cyclophosphamide-treated mice had a significantly reduced bodyweight and thymus and spleen coefficients [42, 43], which was consistent with our results.

Cytokines always reflects immune state to some extent [44]. Once immune system was damaged, the cytokines will be regulated immediately against intruder [45]. IL-2 regulates immunity via affecting and regulating T lymphocytes, which involves the whole life cycle of Th1 cells and Th2 cells. Besides, cyclophosphamide could cause Th1/Th2 bias [46]; thus, the level of IL-2 was detected in serum. Compared with the IL-2 level in the CY group, CPP significantly increased IL-2 contents. IL-6 involved the development and maturation of B cell to plasma cells and sustained antibody production [47–50]. IL-6 regulates inflammation and immune response as a mediator and warner [51]; therefore, IL-6 plays an important role in the immune system. In the present study, cyclophosphamide significantly decreased the IL-6 level, while CPP treatment upregulated the IL-6 level notably. IFN-*γ* plays an important role in immune response both in innate and acquired immune, especially in the early stage of immunity [52], which exhibited immunoregulation during blepharon conjunctivitis in brown Norway rats at the phase of induction [53]. Compared with the IFN-*γ* level in the control group, cyclophosphamide significantly decreased and then IFN-*γ* content, which was recovered by CPP treatment. TNF-*α* mediates innate immunity in the early stage of immune system damaged [54], and it also exhibited the ability to regulate leukocyte to mediate organism's immune state [45]; therefore, the level of TNF-*α* reflects immune state to some extent. The TNF-*α* content was decreased by cyclophosphamide which was also enhanced by CPP. In the present study, the levels of the test cytokines in serum were consistent with the mRNA levels in the spleen obtained by RT-PCR assay.

The NF-*κ*B signaling pathway is essential to rapid induction of expressions of acute-phase antimicrobial defense genes against pathogens, and the functions include regulating lymphoid organ development, B cell survival and maturation, and the differentiation of osteoclasts [55]. In the present study, cyclophosphamide treatment significantly activated the NF-*κ*B signaling pathway, while CPP decreased the expression of NF-*κ*B p65. It indicated that CPP inhibited the immunosuppression induced by cyclophosphamide, which was consistent with the function of NF-*κ*B in the immune system [56]. Toll-like receptors (TLRs) inducing proinflammatory response is the first line of host defense against external invasion and protecting host [57, 58]. In all TLRs, TLR4 exhibited unique ability to identify pathogen-associated molecular patterns (PAMPs) from multiple types of pathogens [58]. In the present study, CY treatment activated the expression of TLR4 which was significantly decreased by CPP, indicating CPP exhibited potent anti-inflammation activity to protect host. MyD88, a common adaptor, was considered as an essential component for activation of all the TLRs in innate immunity [57]. Previous studies indicated that there were few productions of inflammatory cytokines against TLR ligands in MyD88 knockout mice [59]. In this study, the expression of MyD88 was increased by cyclophosphamide, which was significantly inhibited by CPP. The variation of MyD88 was consist with the change of the TLR4 level.

## 5. Conclusion

CPP showed potent immunoregulatory ability in immunosuppressive mice through regulating NF-*κ*B and TLRs signaling pathways. CPP exhibited the potential for treating immunosuppression, especially induced by cyclophosphamide.

## Figures and Tables

**Table 1:** Primers.

Gene	Forward primer	Reverse primer
IL-2	ATTTGAGTGCCAATTCGATG	AGCCTTATGTGTTGTAAGCA
IL-6	AATGTCGAGGCTGTGCAGATTAGTAC	GGGTGGTGGCTTTGTCTGGATTC
IFN-*γ*	TCAGGTGGCATAGATGTGGAAGAA	TGGCTCTGCAGGATTTTCATG
TNF-*α*	GCACTGAGAGCATGATCCGAGAC	CGACCAGGAGGAAGGAGAAGAGG
*β*-Actin	GCGCTTTTGACTCAGGATTT	CCTGGGCCATTCAGAAATTA

## Data Availability

The data used to support the findings of this study are included within the article.

## References

[1] Sun Y., Lenon G. B., Yang A. W. H. (2019). Phellodendri Cortex: A Phytochemical, Pharmacological, and Pharmacokinetic Review. *Evidence-based complementary and alternative medicine: eCAM*.

[2] Jiang Q. J., Chen W., Dan H. (2016). Phellodendri Cortex Extract Relaxes Airway Smooth Muscle. *Evidence-based complementary and alternative medicine: eCAM*.

[3] Li S., Liu C., Guo L. (2014). Ultrafiltration liquid chromatography combined with high-speed countercurrent chromatography for screening and isolating potential *α*-glucosidase and xanthine oxidase inhibitors from Cortex *Phellodendri*. *Journal of Separation Science*.

[4] Lee Y.-M., Kim H., Hong E.-K., Kang B.-H., Kim S.-J. (2000). Water extract of 1:1 mixture of Phellodendron cortex and Aralia cortex has inhibitory effects on oxidative stress in kidney of diabetic rats. *Journal of Ethnopharmacology*.

[5] Mao Y.-F., Li Y.-Q., Zong L., You X.-M., Lin F.-Q., Jiang L. (2010). Methanol extract ofPhellodendri cortexalleviates lipopolysaccharide-induced acute airway inflammation in mice. *Immunopharmacology and Immunotoxicology*.

[6] Xian Y.-F., Mao Q.-Q., Ip S.-P., Lin Z.-X., Che C.-T. (2011). Comparison on the anti-inflammatory effect of cortex Phellodendri chinensis and cortex Phellodendri Amurensis in 12-O-tetradecanoyl-phorbol-13-acetate-induced ear edema in mice. *Journal of Ethnopharmacology*.

[7] Chen G., Li K.-K., Fung C.-H. (2014). Er-Miao-San, a traditional herbal formula containing Rhizoma Atractylodis and Cortex Phellodendri inhibits inflammatory mediators in LPS-stimulated RAW264.7 macrophages through inhibition of NF-*κ*B pathway and MAPKs activation. *Journal of Ethnopharmacology*.

[8] Uchiyama T., Kamikawa H., Ogita Z. (1989). Anti-ulcer effect of extract from Phellodendri cortex. *Yakugaku Zasshi*.

[9] Jung H. W., Jin G.-Z., Kim S. Y., Kim Y. S., Park Y.-K. (2009). Neuroprotective effect of methanol extract of *Phellodendri* Cortex against 1-methyl-4-phenylpyridinium (MPP+)-induced apoptosis in PC-12 cells. *Cell Biology International*.

[10] Xu X., Pan Y., Xu B. (2020). Effects of Cortex *Phellodendri* extract on post‐weaning piglets diarrhoea. *Veterinary medicine and science*.

[11] Park J.-I., Shim J.-K., Do J.-W. (1999). Immune-stimulating properties of polysaccharides from *Phellodendri* cortex (Hwangbek). *Glycoconjugate Journal*.

[12] Kim H.-Y., Shin H.-S., Park H. (2008). In vitro inhibition of coronavirus replications by the traditionally used medicinal herbal extracts, *Cimicifuga rhizoma*, *Meliae cortex*, *Coptidis rhizoma*, and *Phellodendron* cortex. *Journal of Clinical Virology*.

[13] Omole J. G., Ayoka O. A., Alabi Q. K. (2018). Protective effect of kolaviron on cyclophosphamide-induced cardiac toxicity in rats. *Journal of Evidence-Based Integrative Medicine*.

[14] Elmariah H., Kasamon Y. L., Zahurak M. (2018). Haploidentical bone marrow transplantation with post-transplant cyclophosphamide using non-first-degree related donors. *Biology of Blood and Marrow Transplantation*.

[15] Iqubal A., Iqubal M. K., Sharma S. (2019). Molecular mechanism involved in cyclophosphamide-induced cardiotoxicity: old drug with a new vision. *Life Sciences*.

[16] Müller L., Di Benedetto S., Pawelec G. (2019). The immune system and its dysregulation with aging. *Sub-cellular biochemistry*.

[17] Dantzer R., O’Connor J. C., Freund G. G., Johnson R. W., Kelley K. W. (2008). From inflammation to sickness and depression: when the immune system subjugates the brain. *Nature Reviews Neuroscience*.

[18] De Heredia F. P., Gómez-Martínez S., Marcos A. (2012). Obesity, inflammation and the immune system. *Proceedings of the Nutrition Society*.

[19] Aoshi T., Koyama S., Kobiyama K., Akira S., Ishii K. J. (2011). Innate and adaptive immune responses to viral infection and vaccination. *Current opinion in virology*.

[20] Vasquez I., Cao T., Hossain A. (2020). Aeromonas salmonicida infection kinetics and protective immune response to vaccination in sablefish (*Anoplopoma fimbria*). *Fish & Shellfish Immunology*.

[21] Blanco J. L., Garcia M. E. (2008). Immune response to fungal infections. *Veterinary Immunology and Immunopathology*.

[22] Pawelec G. J. I. (2012). Hallmarks of human “immunosenescence”: adaptation or dysregulation?. *Immunity and Ageing*.

[23] Ludmila M., Graham P. (2014). Aging and immunity – impact of behavioral intervention. *Journal of Brain Behavior & Immunity*.

[24] Moon C. K., Sim K. S., Lee S. H. (1983). Antitumor activity of some phytobased polysaccharides and their effects on the immune function. *Archives of Pharmacal Research*.

[25] Nazeam J. A., Gad H. A., Esmat A., El-Hefnawy H. M., Singab A.-N. B. (2017). Aloe arborescensPolysaccharides:in VitroImmunomodulation and potential cytotoxic activity. *Journal of Medicinal Food*.

[26] Liu Y., Sun Y., Huang G. (2018). Preparation and antioxidant activities of important traditional plant polysaccharides. *International Journal of Biological Macromolecules*.

[27] Huang G., Mei X., Hu J. (2017). The antioxidant activities of natural polysaccharides. *Current Drug Targets*.

[28] Liu L., Li M., Yu M. (2019). Natural polysaccharides exhibit anti-tumor activity by targeting gut microbiota. *International Journal of Biological Macromolecules*.

[29] Liu M., Li S., Wang X. (2018). Characterization, anti-oxidation and anti-inflammation of polysaccharides by Hypsizygus marmoreus against LPS-induced toxicity on lung. *International Journal of Biological Macromolecules*.

[30] Dong N., Li X., Xue C. (2020). Astragalus polysaccharides alleviates LPS-induced inflammation via the NF-*κ*B/MAPK signaling pathway. *Journal of Cellular Physiology*.

[31] Liu Z.-h., Niu F.-j., XIE Y.-x. (2020). A review: natural polysaccharides from medicinal plants and microorganisms and their anti-herpetic mechanism. *Biomedicine & Pharmacotherapy*.

[32] Chen L., Huang G. (2018). The antiviral activity of polysaccharides and their derivatives. *International Journal of Biological Macromolecules*.

[33] Wang W., Xue C., Mao X. (2020). Radioprotective effects and mechanisms of animal, plant and microbial polysaccharides. *International Journal of Biological Macromolecules*.

[34] Yin M., Zhang Y., Li H. (2019). Advances in research on immunoregulation of macrophages by plant polysaccharides. *Frontiers in Immunology*.

[35] Karnati H. K., Pasupuleti S. R., Kandi R. (2015). TLR-4 signalling pathway: MyD88 independent pathway up-regulation in chicken breeds upon LPS treatment. *Veterinary Research Communications*.

[36] Zhang Y., Feng G., Ni Y., Ruqian Z. (2013). LPS-induced inflammation in the chicken is associated with CCAAT/enhancer binding protein beta-mediated fat mass and obesity associated gene down-regulation in the liver but not hypothalamus. *BMC Veterinary Research*.

[37] Jeurissen S. H. M., Claassen E., Janse E. M. (1992). Histological and functional differentiation of non-lymphoid cells in the chicken spleen[J]. *Immunology*.

[38] Poujol F., Monneret G., Pachot A., Textoris J., Venet F. (2015). Altered T lymphocyte proliferation upon lipopolysaccharide challenge ex vivo. *PLoS One*.

[39] Xie X., Shen W., Zhou Y. (2020). Characterization of a polysaccharide from Eupolyphaga sinensis walker and its effective antitumor activity via lymphocyte activation. *International Journal of Biological Macromolecules*.

[40] Huang R., Shen M., Yu Y., Liu X., Xie J. (2020). Physicochemical characterization and immunomodulatory activity of sulfated Chinese yam polysaccharide. *International Journal of Biological Macromolecules*.

[41] Zhao X., Liu Q., Sun H., Hu Y., Wang Z. (2017). Chronic systemic toxicity study of copper intrauterine devices in female wistar rats. *Medical Science Monitor*.

[42] Zhou Y., Chen X., Yi R. (2018). Immunomodulatory effect of tremella polysaccharides against cyclophosphamide-induced immunosuppression in mice. *Molecules*.

[43] Shuai W., Shuo H., Qianhong Y. (2018). Prevention of cyclophosphamide-induced immunosuppression in mice with the antimicrobial peptide sublancin. *Journal of Immunology Research*.

[44] Cervi L., Cejas H., Masih D. T. (2001). Cytokines involved in the immunosuppressor period in experimental fasciolosis in rats. *International Journal for Parasitology*.

[45] Sedgwick J. D., Riminton D. S., Cyster J. G., Körner H. (2000). Tumor necrosis factor: a master-regulator of leukocyte movement. *Immunology Today*.

[46] Adams D. O., Hamilton T. A. (1984). The cell biology of macrophage activation. *Annual Review of Immunology*.

[47] Hunter C. A., Jones S. A. (2015). IL-6 as a keystone cytokine in health and disease. *Nature Immunology*.

[48] Liu X., Jones G. W., Choy E. H., Jones S. A. (2016). The biology behind interleukin-6 targeted interventions. *Current Opinion in Rheumatology*.

[49] Chavele K. M., Merry E., Ehrenstein M. R. (2015). Cutting edge: circulating plasmablasts induce the differentiation of human T follicular helper cells via IL-6 production. *The Journal of Immunology (Baltimore)*.

[50] Barr T. A., Shen P., Brown S. (2012). B cell depletion therapy ameliorates autoimmune disease through ablation of IL-6-producing B cells. *Journal of Experimental Medicine*.

[51] Tanaka T., Narazaki M., Kishimoto T. (2014). IL-6 in inflammation, immunity, and disease. *Cold Spring Harbor perspectives in biology*.

[52] Thäle C., Kiderlen A. F. (2005). Sources of interferon-gamma (IFN-gamma) in early immune response to Listeria monocytogenes. *Immunobiology*.

[53] Fukushima A., Fukata K., Ozaki A., Kuroda N., Enzan H., Ueno H. (2002). Exertion of the suppressive effects of IFN-gamma on experimental immune mediated blepharoconjunctivitis in Brown Norway rats during the induction phase but not the effector phase. *British Journal of Ophthalmology*.

[54] Beutler B., Cerami A. (1989). The biology of cachectin/TNF -- A primary mediator of the host response. *Annual Review of Immunology*.

[55] Sun S.-C. (2017). The non-canonical NF-*κ*B pathway in immunity and inflammation. *Nature Reviews Immunology*.

[56] Li Q., Verma I. M. (2002). NF-*κ*B regulation in the immune system. *Nature Reviews Immunology*.

[57] Takeda K., Akira S. (2004). TLR signaling pathways. *Seminars in Immunology*.

[58] Mukherjee S., Huda S., Sinha Babu S. P. (2019). Toll-like receptor polymorphism in host immune response to infectious diseases: a review. *Scandinavian Journal of Immunology*.

[59] Doyle S. E., Vaidya S. A., O’Connell R. (2002). IRF3 mediates a TLR3/TLR4-specific antiviral gene program. *Immunity*.

